# Acute Cytomegalovirus Infection as a Rare Cause of Portal Vein Thrombosis with Small Bowel Infarction in an Immunocompetent Patient

**DOI:** 10.5334/jbr-btr.1251

**Published:** 2017-03-29

**Authors:** Arnout Vael, Hendrik Degryse, Peter Bracke

**Affiliations:** 1 University of Antwerp, BE; 2 AZ KLINA, BE

**Keywords:** Cytomegalovirus thrombosis, Portal vein thrombosis, CT, Small bowel infarction, CT

## Abstract

Portal vein thrombosis (PVT) refers to thrombosis that develops in the trunk of the portal vein including its right and left intrahepatic branches and may even extend to the splenic or superior mesenteric veins. PVT due to Cytomegalovirus (CMV) infection is a rare complication, scarcely described in English literature. We present a case of a 58-year-old immunocompetent patient with PVT and small bowel ischemia.

A 58-year-old immunocompetent female presented to the emergency department with abdominal discomfort, fever, and increasing chronic fatigue. Cytomegalovirus (CMV) IgM antibodies had already been tested by her general practitioner, and the result was consistent with an acute CMV infection. The treatment was symptomatic and consisted of analgesics and antipyretics.

Five hours later, the patient was readmitted to the emergency department with severe abdominal pain. Her clinical examination revealed abdominal defense, percussion pain, and rebound tenderness. Blood tests showed disturbed liver function and confirmed CMV infection. Abdominal ultrasound showed slight splenomegaly, but the clinical degradation with development of an acute abdomen required prompt contrast-enhanced abdominal CT, which demonstrated thrombosis of the superior mesenteric vein, the right portal vein, part of the left portal vein, and the distal splenic vein (Figures 1, 2, 3). Diffuse hypodense wall thickening of an extensive segment of small bowel was also found, which is consistent with venous ischemia, with no obvious signs of perforation or necrosis (Figure 3). Further investigations were undertaken to exclude hematological, autoimmune, or neoplastic cause for the thrombosis.

**Figure 1:**
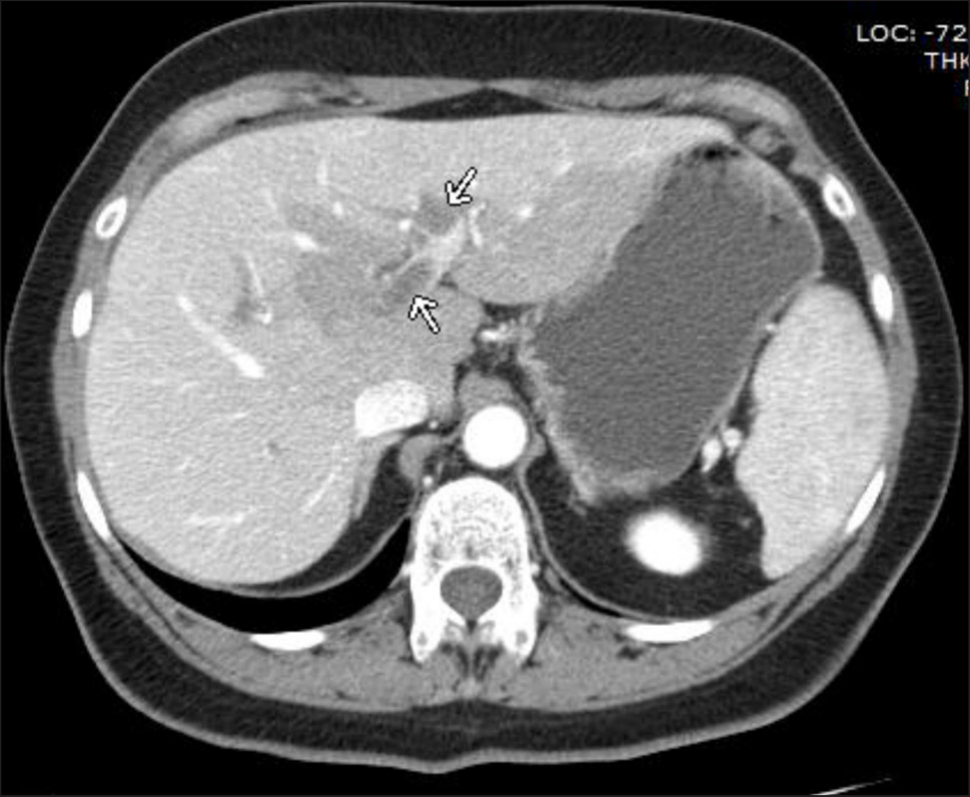
Axial CT image showing the thrombi within the portal veins (arrows).

**Figure 2:**
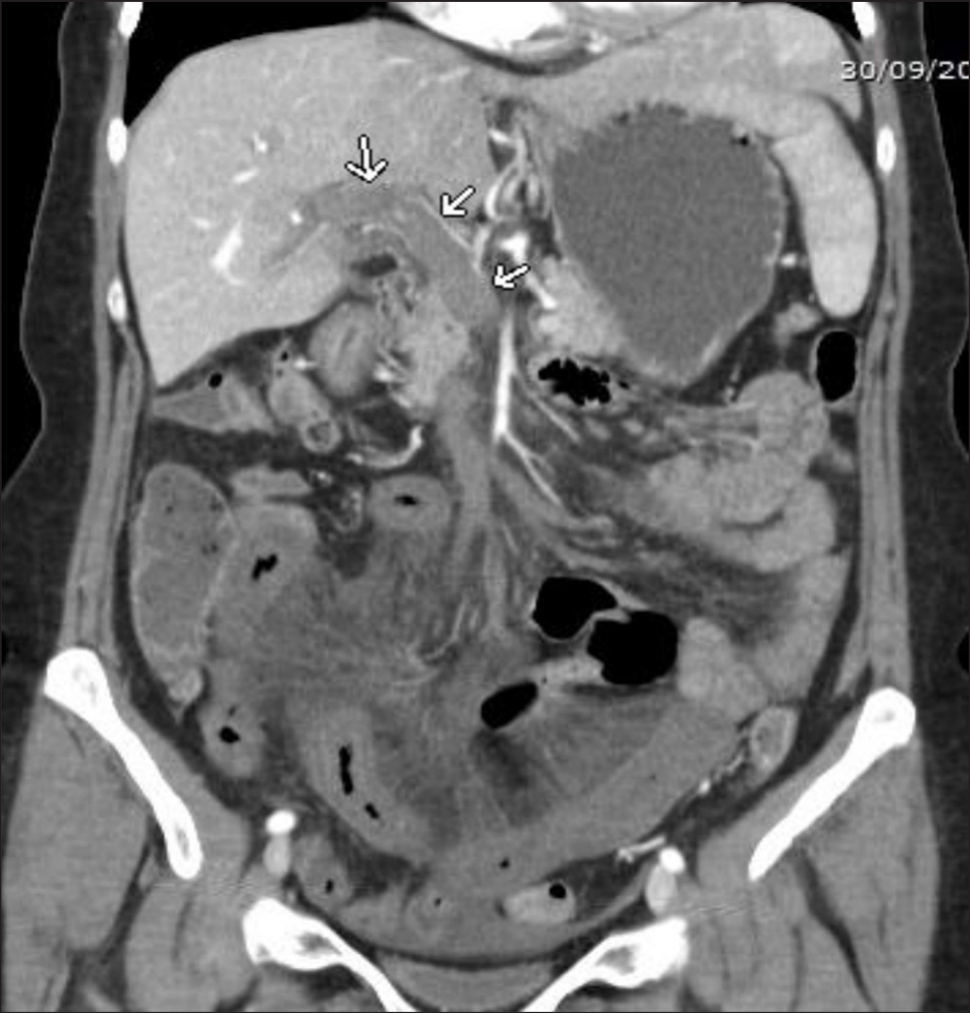
Coronal image showing the extensive portal vein thrombosis (arrows).

**Figure 3:**
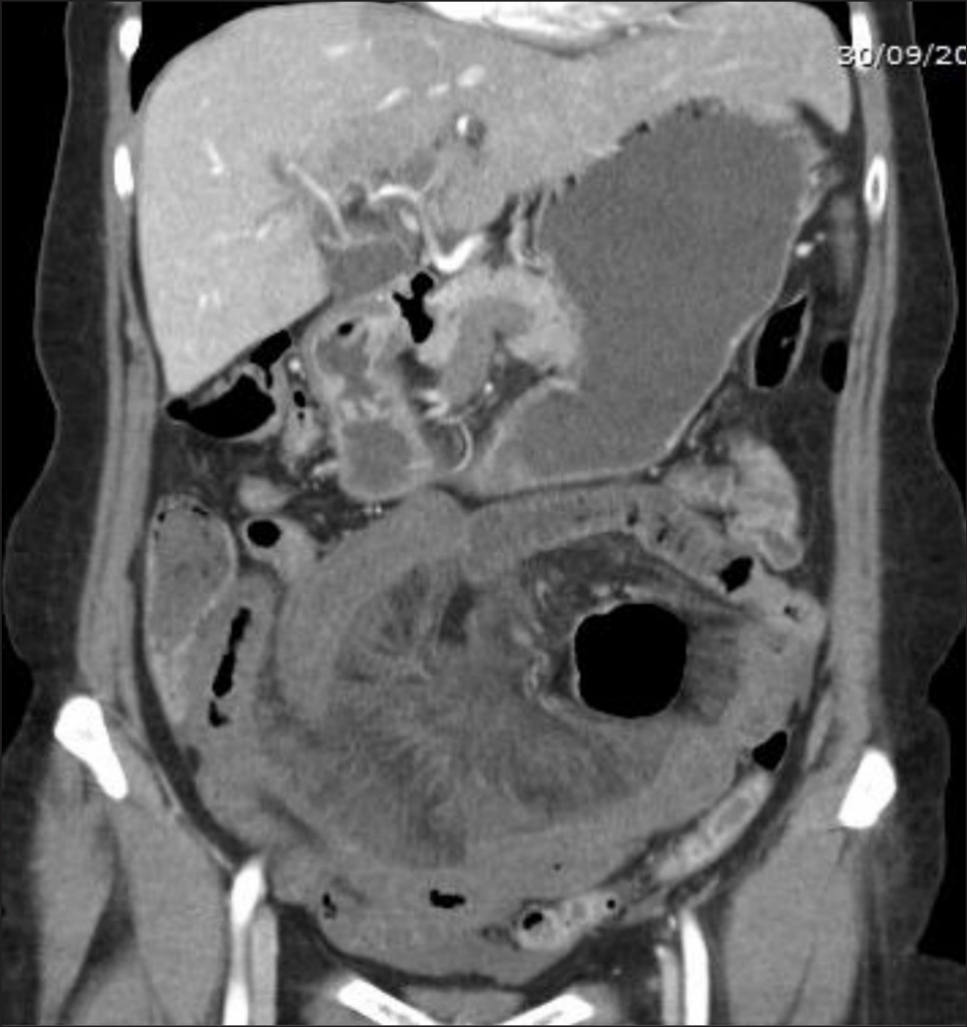
Coronal image demonstrating the venous infarction of the small bowel in the lower abdomen.

The patient was transferred to the nearest University Hospital for systemic thrombolysis (Actilyse; a bolus of 10 mg followed by a continuous infusion of 90 mg in two hours). However, it had to be discontinued due to a large cervical hematoma at the central line site. Treatment with heparin was tried after the bleeding stopped, but it had to be discontinued due to digestive tract bleeding. Gastroscopy excluded esophageal varices and gastric ulcers.

The patient developed a secondary episode of acute abdomen with peritonitis due to secondary small bowel necrosis. Surgical resection of the small bowel and an extensive right hemicolectomy was performed (80–100 cm remaining bowel). The patient was discharged after three weeks.

## Comment

Portal vein thrombosis (PVT) usually occurs in association with cirrhosis or malignancy of the liver. There are however non-cirrhotic cases based on one or more features of Virchow’s triad: reduced portal blood flow, vascular endothelial injury, or a hypercoagulable state. Venous thromboembolism due to acute CMV infection is a well-known complication in immunocompromised patients. However, in immunocompetent patients, it is often underestimated or not considered. According to a recent prospective study by Paran et al., the reported incidence rate is 6.9 percent [1]. Our patient, who had only a recent CMV seroconversion as shown by the serological and PCR testing, presented acutely with abdominal pain. The diagnosis of extensive portal, superior mesenteric, and splenic vein thrombosis combined with small bowel ischemia was promptly confirmed by an emergency abdominal contrast-enhanced CT.

A contrast-enhanced CT scan is the gold standard to promptly diagnose PVT. Arterial phase usually provides little information; the diagnosis can only be reliably made during the portal venous phase. CT findings include demonstration of the thrombus itself, presenting as a well-defined intraluminal filling defect with central low attenuation, which may be surrounded by enhanced venous walls. Accompanying collateral circulation, engorgement of mesenteric veins, and mesenteric edema may be present. Bowel wall thickening is the most common manifestation of venous bowel ischemia. The combination of low attenuation in the superior mesenteric vein, thickening of the small bowel wall, and the presence of ascites make bowel infarction very likely.

Various causative mechanisms have been considered as the reason for CMV-associated thrombosis. However, the currently most accepted theory is that CMV transiently induces production of antiphospholipid antibodies [2]. Phospholipids are present in the membranes that form the surface of cells, including blood cells and endothelial cells. The development of antibodies against proteins that are attached to phospholipids may increase the risk of developing blood clots in the veins or arteries and may also cause an increased risk of miscarriage or stillbirth among pregnant women.

Our case illustrates that venous thromboembolism caused by an acute CMV infection may occur in immunocompetent patients. The importance of a contrast-enhanced CT scan to allow for early diagnosis and therapy is illustrated.
